# Inflammatory predictors of Post-COVID fatigue

**DOI:** 10.1016/j.bbih.2025.101109

**Published:** 2025-09-17

**Authors:** A. Nuber-Champier, G. Breville, P. Voruz, I. Jacot de Alcântara, P.H. Lalive, G. Allali, L. Benzakour, K.-O. Lövblad, O. Braillard, M. Nehme, M. Coen, J. Serratrice, J.-L. Reny, J. Pugin, I. Guessous, B.N. Landis, A. Cionca, F. Assal, J.A. Péron

**Affiliations:** a Clinical and Experimental Neuropsychology Laboratory, Faculty of Psychology, University of Geneva, Geneva, Switzerland; b Neurology Division, Geneva University Hospitals, Switzerland; c Department of Neurology, Perelman School of Medicine, University of Pennsylvania, Philadelphia, USA; d Neurosurgery Department, Geneva University Hospitals, Switzerland; e Faculty of Medicine, University of Geneva, Switzerland; f Leenaards Memory Center, Lausanne University Hospital and University of Lausanne, Lausanne, Switzerland; g Psychiatry Department, Geneva University Hospitals, Switzerland; h Diagnostic and Interventional Neuroradiology Department, Geneva University Hospitals, Switzerland; i Division and Department of Primary Care, Geneva University Hospitals, Switzerland; j Division of General Internal Medicine, Department of Medicine, Geneva University Hospitals and Geneva University, Switzerland; k Intensive Care Department, Geneva University Hospitals, Switzerland; l Rhinology-Olfactology Unit, Otorhinolaryngology Department, Geneva University Hospitals, Switzerland; m École Polytechnique Fédérale de Lausanne (EPFL), School of Engineering, Neuro-X Institute, Lausanne, Switzerland

**Keywords:** SARS-CoV-2, Fatigue, Immunity, COVID-19, Immunology, Long COVID, Inflammation

## Abstract

The biological mechanisms underlying objective and subjective fatigue in post-COVID syndrome remain unclear. This study investigates whether immune responses during the acute phase of SARS-CoV-2 infection predict fatigue dimensions 6–9 months post-infection. We analyzed serum immune markers from 54 hospitalized patients (mean age: 58.69 ± 10.90 yrs; female: 31 %) and assessed their association with chronic fatigue using general linear mixed models. Elevated levels of IL-1RA, IFNγ, TNFα, and monocyte percentage during acute infection predicted increased physical and total fatigue. Additionally, higher TNFα levels (r = −0.40, *p* = .019) correlated with reduced awareness of cognitive fatigue. These findings highlight the role of acute inflammation in the persistence of post-COVID fatigue.

## Highlights

• TNFα levels are associated with fatigue awareness.
• Monocyte percentage predicts subjective fatigue.
• Different inflammatory markers are associated with different dimensions of fatigue.

## 1 Introduction

Fatigue is a common clinical complaint across various pathologies (Seeβle et al., 2022; Mazza et al., 2022), particularly in post-COVID syndrome (Davis et al., 2023). It is one of the most persistent symptoms, lasting beyond a year after SARS-CoV-2 infection (Seeβle et al., 2022). Studies have linked post-COVID fatigue to structural brain changes in the thalamus and basal ganglia (Heine et al., 2023; Diez-Cirarda et al., 2024; Hosp et al., 2024) and highlighted similarities with myalgic encephalomyelitis/chronic fatigue syndrome (ME/CFS) (Bateman et al.), particularly regarding neuroinflammatory responses. Elevated levels of cytokines such as IL-1, TNFα, and IFNγ, along with immune cellular variations, suggest a role for systemic immune dysregulation in the persistence of fatigue (Morris and Maes, 2013).

Monocytes and cytokines have emerged as key markers in this process (Ceban et al., 2022; Azzolino and Cesari, 2022). Persistent symptoms, including fatigue, have been associated with monocyte-platelet aggregates and elevated plasma cytokines beyond one-year post-infection (Voruz et al., 2022a; Davis et al., 2023; Azzolino and Cesari, 2022). While hyperinflammation during the acute phase is frequently implicated in post-COVID pathophysiology, fewer studies have examined the continuum of immune response and its potential long-term effects, including low immune activation during the acute phase (Mazzoni et al.; Nuber-Champier et al., 2022).

Interestingly, discrepancies exist between subjective and objective fatigue in post-COVID patients (Ceban et al., 2022). Subjective fatigue is self-reported, while objective fatigue is measured through attentional functions, independent of self-perception. Also, some studies suggest the existence of distinct post-COVID phenotypes that can be situated on a self-awareness continuum (Voruz et al., 2022a; Nuber-Champier et al., 2023). Prior research has shown that anosognosia of cognitive deficits, where patients are unaware of their neuropsychological impairments, is linked to increased monocytes and TNFα levels during the acute phase, as well as poorer cognitive performance months later (Nuber-Champier et al., 2022, 2023). This raises the question of whether a similar lack of awareness exists for fatigue and whether it defines a distinct patient trajectory with cognitive or psychiatric implications (Voruz et al., 2024). This phenomenon of (un-)awareness of disorders and these inflammatory variations, particularly linked to TNFα, IL-1, but also IL-6 and IL-8 levels, also suggests similarities with neurodegenerative trajectories (Rubio-Perez et al., 2012).

Despite fatigue being a multidimensional phenomenon (physical, social, cognitive, and psychological), most studies have treated it as a single entity. Moreover, few have explored how immune markers during the acute phase predict different fatigue dimensions months later.

This study aims to (1) assess the predictive relationship between acute-phase inflammatory markers (IL-1β, IL-1RA, IFNγ, TNFα, and monocytes) and post-COVID fatigue dimensions at 6–9 months, and (2) investigate whether acute immune response influences awareness of cognitive fatigue. Finally, (as ad hoc analyses), this study aims to (3) explore the associations between inflammatory markers (IL-6, IL-8 and other leukocyte subsets including lymphocytes, neutrophils, eosinophils and basophils) and subjective fatigue and awareness of cognitive fatigue, and to (4) determine differences in intergroup inflammatory concentrations (fatigued vs. non-fatigued) as well as to (5) evaluate fatigue levels by stratifying groups according to inflammation level (low vs. moderate vs. high inflammation). We hypothesize that higher levels of monocytes and pro-inflammatory cytokines during acute COVID-19 will be associated with greater fatigue in all dimensions and that acute inflammation will predict reduced awareness of cognitive fatigue (Monje and Iwasaki, 2022; Taquet et al., 2023).

## 2 Method

### 2.1 General procedure

We analyzed data from the COVID-COG cohort, including innate immunity and cytokines measured during the acute phase, as well as objective and subjective fatigue assessed 6–9 months post-infection. A self-appraisal discrepancy score was calculated to quantify fatigue perception. Finally, we examined the association and predictive value of acute-phase immune markers on different dimensions of chronic fatigue. The study was conducted in accordance with the Declaration of Helsinki and approved by the Cantonal Ethics Committee of Geneva (CER-02186).

### 2.2 COVID-COG cohort

The COVID-COG cohort (Voruz et al., 2022b) consists of 121 patients selected based on strict criteria, excluding those with prior neurological, psychiatric, oncological, or neurodevelopmental conditions, as well as pregnant individuals and those over 80 years old. SARS-CoV-2 infection was confirmed via PCR and/or serology (acute infections occurred between March 2020 and May 2021 (Voruz et al., 2024)). Patients were categorized into three groups: i) those in intensive care with mechanical ventilation (N = 24), ii) hospitalized without ventilation (N = 48), and iii) non-hospitalized (N = 49), with comparable socio-demographic characteristics between the three groups. All participants underwent comprehensive neuropsychological testing and psychiatric assessments 6–9 months post-infection. Evaluations were conducted before the rollout of COVID-19 vaccination.

### 2.3 Participants included in the study

**Fig. 1:**
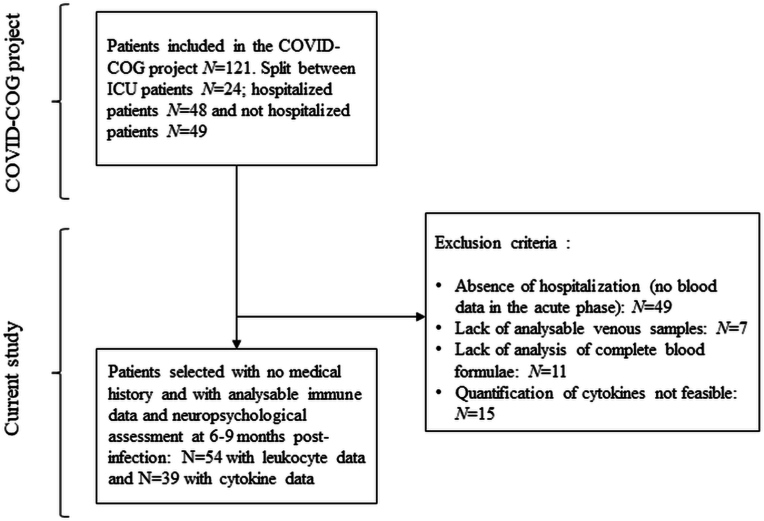
Study flowchart.

From the COVID-COG cohort, which initially consisted of 121 patients, we retained 54 hospitalized patients (*N* = 32 in conventional care and *N* = 22 in intensive care) with leukocyte and fatigue data (see Table 1). Of these 54 patients, 39 had samples that could be analyzed for cytokine quantification (see Fig. 1). Retrospective analysis of markers presents in the acute phase of SARS-CoV-2 infection (between March 2020 and May 2021) in patients in the COVID-COG cohort included white blood cell distribution and concentrations of TNFα, IL-1RA, IL-1β, IL-6, IL-8, IFNγ, G-CSF and GM-CSF (Nuber-Champier et al., 2022, 2023). In this study, we focused on the distribution of white blood cells and the concentrations of TNFα, IL-1RA, IL-1β, IL-6, IL-8 and IFNγ. In our analyses combining inflammatory data and fatigue data, 37 patients had both cytokine data and subjective fatigue data, of whom 33 had data for calculating cognitive fatigue awareness. In terms of leukocyte distribution, 54 patients had both inflammatory data and subjective fatigue scores. Of these, 47 patients had data for calculating cognitive fatigue awareness.

**Table 1:** Socio-demographic and clinical data from the sample of patients assessed 6–9 months after SARS-CoV-2 infection.

	Patients included in the study
	*N* = 54
Mean age in years (±SD)	58.69 (±10.90)
Education level (1/2/3)	2/18/34
Sex (F/M)	17/37
Number of patients who required intermediate/intensive care in the acute phase	32/22
Mean days of hospitalization (±SD)	21.98 (±25.29)
Mean days between positive RT-PCR test and collection of immunological data (±SD)	1.98 (±3.62)
Diabetes (Yes/No)	8/46
History of respiratory disorders (Yes/No)	9/45
History of cardiovascular disorders (Yes/No)	10/44
History of neurological disorders (Yes/No)	0/54
History of psychiatric disorders (Yes/No)	2/52^a^
History of cancer (Yes/No)	0/54
History of severe immunosuppression (Yes/No)	0/54
History of developmental disorders (Yes/No)	0/54

*Note.* Education level: 1 = compulsory schooling, 2 = post-compulsory schooling, and 3 = university degree or equivalent. RT-PCR: reverse transcription polymerase chain reaction; SD: standard deviation; Sex F: female and M: mal. Types of history of respiratory disorders: asthma, chronic bronchitis; Types of history of cardiovascular disorders: previous infarction, valve pathology, atrial pathology and heart failure.

a Types of history of psychiatric disorders: minor depressive episodes more than 10 years ago.

### 2.4 Measurements of fatigue

*Subjective fatigue.* We assessed subjective fatigue sub-dimensions using the French version of the EMIF-SEP questionnaire (Debouverie et al., 2007), a validated 40-item scale covering cognitive (10 items), physical (13 items), social (13 items), and psychological (4 items) fatigue. Physical fatigue refers to bodily exhaustion limiting daily activities, while cognitive fatigue reflects reduced mental capacity (e.g., concentration, memory). Psychological and social fatigue involve emotional distress, irritability, discouragement, isolation, or difficulty maintaining social interactions. Raw scores were converted into percentages for statistical comparability, with higher percentages indicating greater subjective fatigue severity. In order to form groups of patients with significantly low or high subjective fatigue levels, we used the standards established by Debouverie et al. (2007) on a population with multiple sclerosis. We calculated Z scores based on the scores we obtained and the means and standard deviations obtained in Debouverie et al. (2007). Given that the population studied by Debouverie et al. (2007) has multiple sclerosis, we chose a non-conservative threshold. In this sense, Z scores below −1 or above 1.25 were classified as low levels of fatigue or excessively fatigued, respectively (Details available in SI 10).

*Objective cognitive fatigue.* Objective cognitive fatigue was assessed using median T scores from the sustained attention subtest of the Test of Attentional Performance (TAP) (Zimmermann and Fimm, 1995). Higher T scores indicates better performance. This test is commonly used to measure fatigue in conditions like multiple sclerosis (Neumann et al., 2014; Claros-Salinas et al., 2013; Weinges et al., 2010). It requires participants to sustain attention for 15 min, responding as quickly as possible when a target stimulus pair of identical shapes or colors appears.

*Discrepancy between subjective and objective cognitive fatigue.* To compare objective and subjective cognitive fatigue measures, we first inverted the subjective fatigue scores by calculating their complementary percentages (e.g., 80 % fatigue became 20 % preservation). This inversion ensured that higher scores consistently indicated less fatigue. We then weighted the subjective scale against the objective scale. The self-appraisal discrepancy (SAD) score was calculated by subtracting the percentage of subjective cognitive fatigue (from EMIF-SEP) from the median T scores of sustained attention (from TAP (Zimmermann and Fimm, 1995)). We identified three patterns: i) Over-awareness: High subjective fatigue (e.g., 90 %, inverted to 10 %) with low objective fatigue (T score 55) results in a positive discrepancy (e.g., 55-10 = 45), indicating heightened subjective perception. ii) Congruent awareness: Matching subjective (e.g., 60 %, inverted to 40 %) and objective fatigue (T score 44) yields a near-zero discrepancy (e.g., 44-40 = 4), indicating alignment between subjective experience and objective measurement. iii) Anosognosia: Low subjective fatigue (e.g., 30 %, inverted to 70 %) despite high objective fatigue (T score 31) produces a negative discrepancy (e.g., 31–70 = −39), indicating unawareness of fatigue. These patterns offer insight into patients' awareness of cognitive fatigue.

### 2.5 Analysis of cytokines and plasma leukocyte distribution

The analysis of cytokines (pg/ml) of TNFα, interleukin (IL)− 1RA, IL-1β, interferon gamma (IFNγ), was made using commercially available multiplex bead immunoassays (Fluorokine MAP Multiplex Human Cytokine Panel, R&D Systems, Minneapolis, USA) and read using a Bioplex 200 Array Reader (Bio-Rad Laboratories, Hercules, CA, USA) and Luminex® xMAP™ technology (Luminex Corporation, Austin, TX, USA). Plasma leukocyte distribution was evaluated with Piccolo Xpress (Sysmex, Switzerland) tools (see Table 2). The short period of time between blood collection, processing (cell analysis on fresh blood), and freezing (plasma) did not result in any sample alteration (< mean 79 h). Reliable value extrapolated beyond the standard range (below or above) for concentrations of TNFα: ≥0.56 pg/mL; IL-6: ≥0.83 pg/mL; IL-8: ≥1.91 pg/mL; IL-1RA: ≤21,454.18 pg/mL; IL-1β: ≥0.08 pg/mL; IFNγ: ≥0.26 pg/mL. Inter-individual biological replicates were calculated for each marker [(sd/mean)∗100]. Finally, a single batch of analysis was performed to limit undesirable batch effects. In order to stratify our population based on inflammation deployed in the acute phase, we calculated the Z scores of the reactions for each cytokine (TNFα, IFNγ, IL-1β, IL-1RA; IL-6, IL-8). We added these Z scores and determined three groups of inflammatory scores: Z < −1; −1<Z > 1; Z > 1. One group had low inflammation (Z < −1), one group had regulated/moderate inflammation (−1<Z > 1) and one group had high inflammation (Z > 1).

**Table 2:** Immune markers of patients with COVID-19 on admission to hospital.

Immune markers	Plasma cytokines concentration (*N* = 39) and leukocyte distribution (*N* = 54) on day 1 of COVID-19 related hospitalization – Median [95 %CI]
TNFα (pg/ml)	3.8 [3.40; 6.05]
IL-1Ra (pg/ml)	4438.97 [4840.99; 8134,89]
IFNγ (pg/ml)	1.65 [0.99; 2.09]
IL-1β (pg/ml)	0.69 [0.49; 1.28]
IL-6 (pg/ml)	12.78 [13.92; 30.60]
IL-8 (pg/ml)	11.86 [7.15; 41.11]
Monocytes %	6.35 [5.34; 7.28]
Monocytes (G/l)	0.37 [0.32; 0.44]
Neutrophils %	75.20 [71.95; 78.23]
Neutrophils (G/l)	4.99 [4.60; 5.98]
Eosinophils %	0.00 [0.10; 0.38]
Eosinophils (G/l)	0.00 [0.006; 0.027]
Basophils %	0.20 [0.14; 0.26]
Basophils (G/l)	0.009 [0.008; 0.01]

*Note.* IFNγ: interferon gamma; IL: interleukin; TNFα: tumor necrosis factor alpha. The percentage corresponds to the percentage of white blood cells.

### 2.6 Statistical power

We set the β type 2 error at 0.80 and, since our hypotheses were formulated in a specific sense, the threshold for the α relationship was set at 0.025. Finally, we estimated a correlation coefficient on the observed relationship between TNFα levels, cell concentrations (monocytes) and post-COVID cognitive symptoms obtained in Nuber-Champier et al. (Nuber-Champier et al., 2023; Voruz et al., 2022a) and Cervia-Hasler et al. (Cervia-Hasler et al., 2024).

#### 2.6.1 Samples for cytokine analysis

The standard normal deviate for α = Z_α_ = 1.9600

The standard normal deviate for β = Z_β_ = 0.8416

C = 0.5 ∗ ln[(1+r)/(1-r)] = 0.6625

Total sample size = N = [(Z_α_+Z_β_)/C]2 + 3 = 21

#### 2.6.2 Samples for cell analysis

The standard normal deviate for α = Z_α_ = 1.9600

The standard normal deviate for β = Z_β_ = 0.8416

C = 0.5 ∗ ln[(1+r)/(1-r)] = 0.4001

Total sample size = N = [(Z_α_+Z_β_)/C]^2^ + 3 = 52

Based on the calculation made by Hulley et al. (2013), the necessary sample size is estimated at 52 participants for the cellular analyses and 21 participants for the cytokine analyses.

### 2.7 Statistical analyses

*Data preprocessing and transformation*. To appropriately handle the immune response data, especially cytokine concentrations approaching zero, we applied a logarithmic transformation.

*Statistical approach and multiple comparison correction*. Given the distribution of behavioral data, non-parametric statistical tests were used. False discovery rate (FDR) corrections were applied to all analyses.

*Association between inflammation during the acute phase and subjective fatigue dimensions 6–*9 months *post-infection*. To test our first hypothesis which posited an association between inflammatory variables (e.g., TNFα, IFNγ, IL-1β, IL-1RA, monocytes) measured during the acute phase and various fatigue dimensions (total, physical, cognitive, social, psychological) at 6–9 months post-infection, we conducted Spearman correlations. Next, to explore the predictive capacity of acute-phase immune variables on these subjective fatigue scores at 6–9 months, we used generalized linear mixed models (GLMM) with gamma distribution, incorporating log-transformed inflammatory marker levels as a fixed variable, and age and sex as random variables. These correlation and prediction analyses were also performed according to hospitalization severity subgroups during the acute phase and are presented in Supplementary Material 1-7.

*Association between immune markers and cognitive fatigue self-awareness*. To assess the association and predictive value of acute-phase immune activity on cognitive fatigue self-awareness, we performed Spearman correlations and generalized linear mixed models (GLMM), using inflammatory markers (e.g., TNFα, IL-1β, monocytes), age, and sex as predictors, in line with previous analyses. Specifically, we examined the associations between white blood cell counts, and concentrations of TNFα, IFNγ, IL-1β, IL-1RA, IL-6, and IL-8 measured during the acute phase, and cognitive fatigue scores (SAD) assessed 6–9 months post-infection, while controlling for age and sex. These correlation and prediction analyses were also performed according to hospitalization severity subgroups during the acute phase and are presented in Supplementary Material 1-7.

*Exploratory analysis by subgroups (inflammation and fatigue) (*ad hoc *analyses)*. Finally, exploratory ad hoc analyses of intergroup differences based on EMIF-SEP Z scores (two groups: low fatigue/excessive fatigue) and inflammation stratification Z scores (three groups: mild inflammation/moderate inflammation/severe inflammation) were performed using Kruskal-Wallis group comparisons. We compared inflammatory marker concentrations according to subgroups for each dimension of subjective fatigue. We also compared the levels of fatigue for each dimension according to inflammatory subgroups.

All analyses were performed with SPSS Statistics version 28.0.1.

## 3 Results

### 3.1 Sociodemographic and clinical data

In this study, we included middle-aged patients hospitalized in intermediate or intensive care, with no relevant medical history before the infection (see Table 1). The results of additional correlation and prediction analyses obtained by subgroup according to the type of hospitalization in the acute phase are available in supplementary material 1-7.

### 3.2 Cytokines plasma levels and leukocyte distribution measured during the acute phase of COVID-19

The levels of immune markers (cytokines and monocytes) are presented in Table 2 in logarithmically untransformed form.

With regard to our samples, we observed significant coefficients of variation (CV) in inter-individual biological replicates of inflammatory responses measured during the acute phase. We observed CVs of 86.4 % for TNFα, 115.5 % for IL-6, 217.0 % for IL-8, 78.3 % for IL-1Ra, 137.7 % for IL-1β, and 109.9 % for IFNγ. Leukocyte subset variability was also substantial, including CVs of 49.6 % for neutrophils, 232.1 % for eosinophils, 116.4 % for basophils, and 60.7 % for monocytes.

### 3.3 Association between plasma cytokines concentration, monocytes % measured in the acute phase of COVID-19 and fatigue dimensions 6–9 months post-infection

a) Total fatigue

**Fig. 2:**
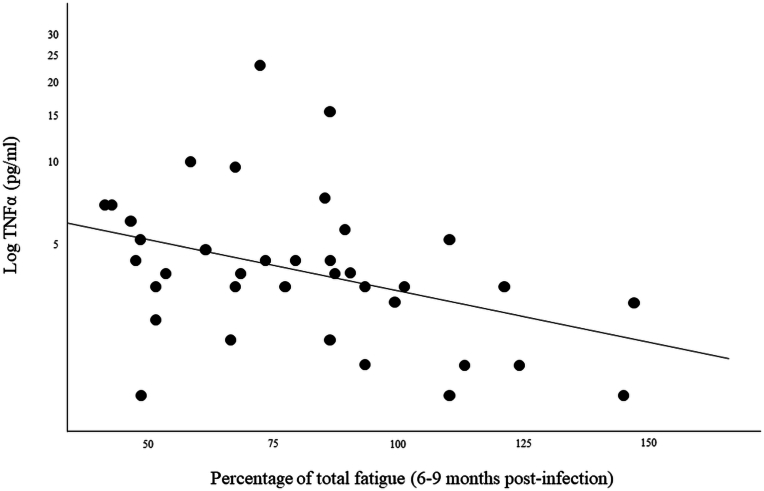
Plasma TNFα levels measured during the acute phase in relation to chronic fatigue percentage.

We observed significant negative associations between total fatigue scores measured 6–9 months post-infection and TNFα levels (r = −0.44; *p* = .006) and also with IL-1RA levels (r = −0.34; *p* = .036) (see Fig. 2) measured during the acute phase.

**Fig. 3:**
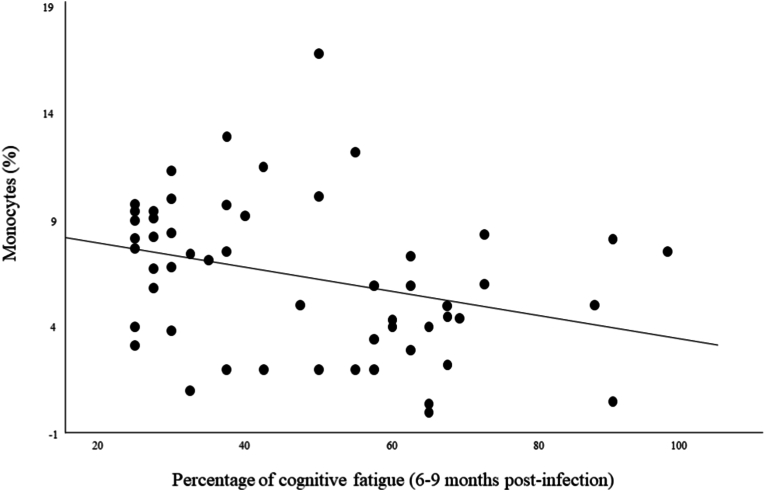
Monocytes percentage measured during the acute phase in relation with chronic cognitive fatigue percentage.

b) Cognitive fatigue
  Blood monocyte percentage among white blood cells measured during the acute phase were significantly negatively associated with cognitive fatigue scores measured 6–9 months post-infection (r = −0.35; *p* = .009) (see Fig. 3).

c) Physical fatigue
  TNFα plasma levels measured during the acute phase were significantly negatively associated with physical fatigue scores measured 6–9 months post-infection (r = −0.42; *p* = .008).

d) Social and psychological fatigue

None of the results were significant. The results are available as supplementary material 8.

### 3.4 Prediction of fatigue dimensions 6–9 months post-infection by inflammation measured during the acute phase of SARS-CoV-2 infection

a) Total fatigue

The model for predicting total fatigue (AIC_c_ = 31.54), which included variables such as sex, age, plasma levels of TNFα, IL-1RA, IL-1β, IFNγ and the percentage of blood monocytes measured during the acute phase, significantly selected IL-1RA (*β* = −0.37; *F* = 4.46; *p* = .044; 95 %CI [−0.73; −0.01]) and IFNγ (*β* = 0.31; *F* = 5.02; *p* = .034; 95 %CI [0.026; 0.59]) plasma levels to predict total fatigue 6–9 months post-infection.

b) Cognitive fatigue

None of the results were significant. The results are available as supplementary material 9.

c) Physical fatigue

There was a significant effect for the predictive ability of IFNγ plasma levels (*β* = 0.40; *F* = 6.39; *p* = .018; 95 %CI [0.076; 0.73]) measured during the acute phase in predicting physical fatigue 6–9 months post-infection (AIC_c_ = 40.29) was observed.

d) Social and psychological fatigue

None of the results were significant. The results are available as supplementary material 9.

### 3.5 Association between immunity measured during the acute phase and awareness of cognitive fatigue 6–9 months post-infection

**Fig. 4:**
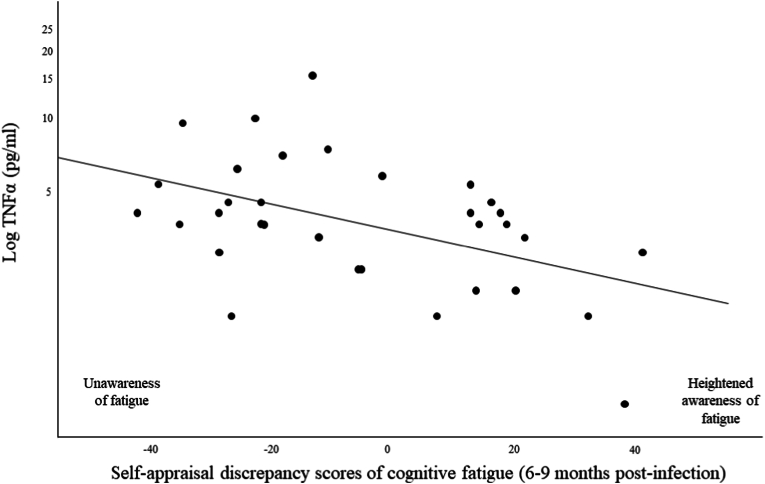
TNFα levels measured during the acute phase in relation with self-appraisal discrepancy scores of cognitive fatigue (6–9 months post-infection). *Note*. Self-appraisal discrepancy of cognitive fatigue (SAD) corresponds to the difference between the objective measures of cognitive fatigue and the subjective measures of cognitive fatigue obtained 6–9 months post-infection. The higher positive the SAD scores, the greater the subjective fatigue symptoms but the lower the objective fatigue symptoms. The lower negative the SAD scores, the lower the subjective fatigue symptoms but the higher the objective fatigue symptoms. Therefore, the lower the SAD values, the greater the patients' anosognosia of their cognitive fatigue. Values around 0 correspond to estimates of fatigue that are adequate between subjective and objective complaints.

We observed a negative correlation between TNFα levels measured during the acute phase and SAD scores of cognitive fatigue obtained 6–9 months post infection (r = −0.40; *p* = .019) (see Fig. 4).

### 3.6 Prediction of immunity measured during the acute phase on awareness of cognitive fatigue 6–9 months post-infection

The regression model concerning the prediction of the SAD scores of cognitive fatigues (AIC_c_ = 34.64), which included variables such as sex, age, levels of TNFα, IL-1RA, IL-1β, IFNγ and the percentage of monocytes measured during the acute phase, was significant for the IL-1RA levels (*β* = −1.57; *F* = 6.12; *p* = .048; 95 %CI [−3.13;-0.17]).

### 3.7 Ad hoc analyses: exploratory results

The exploratory results of the inflammatory differences between the different fatigue groups obtained by Z score and inflammation stratification, and the associations between IL-6/IL-8 and other leukocyte subsets markers and subjective fatigue scores are available in SI 11 to SI 13.

## 4 Discussion

In this study, we examined the relationship between immune response during the acute phase of COVID-19 and fatigue 6–9 months post-infection in previously hospitalized individuals. Our findings reveal significant negative associations between early inflammatory markers and long-term fatigue, suggesting that immune response during the acute phase may shape distinct post-COVID fatigue trajectories.

Lower TNFα and IL-1RA levels during the acute phase were linked to higher total fatigue scores, with TNFα and IL-6 also negatively associated with physical fatigue. Monocyte percentage showed an inverse relationship with cognitive fatigue, whereas neutrophil counts were positively associated. Moreover, IL-1RA and IFNγ plasma levels during acute infection significantly predicted total fatigue, while IFNγ alone was a predictor of physical fatigue. These findings suggest that both pro- and anti-inflammatory responses influence long-term fatigue development. The action and management of neutrophils and monocytes and associated signaling pathways could lead to different fatigue trajectories (Aspler et al., 2008; Paludan and Mogensen, 2022). Although other studies have reported a significant increase in the number of neutrophils and monocytes in patients with chronic fatigue (Saito et al., 2024), our results also support the need for further research on the different dimensions of fatigue and its subjective and objective nature, in relation to inflammatory phenomena. In the future, it will be necessary to clarify the different stages of maturation of neutrophils and monocytes and the signaling pathways associated with post-COVID fatigue.

We also found that higher TNFα levels were associated with reduced self-awareness of cognitive fatigue, suggesting that inflammation may impair symptom perception. Similarly, IL-1RA levels predicted fatigue awareness, highlighting the role of early immune regulation in shaping subjective experience of fatigue (Nuber-Champier et al., 2023, 2024). This suggests that inflammatory markers such as TNFα and monocytes could serve as potential biomarkers for predicting post-COVID trajectories. These results support the hypothesis that immuno-cognitive mechanisms contribute to distinct post-COVID phenotypes (Hartung et al., 2022).

Our findings challenge a linear inflammation–fatigue model, instead pointing to an inverted-U relationship: both insufficient and excessive immune responses may result in fatigue, though through different pathways. Patients with heightened acute inflammation appear more prone to cognitive sequelae and reduced fatigue awareness, whereas those with lower inflammation tend to report higher subjective fatigue. This aligns with prior research indicating that post-viral fatigue syndromes involve complex immune mechanisms beyond direct viral effects (Nuber-Champier et al., 2023; Taquet et al., 2023). Our observations and this hypothesis at the cognitive and immune levels are in line with the results obtained by Kervevan et al. (2023) according to whom two phenotypes were observed in the long COVID. On the one hand, patients with strong and durable immune responses and on the other, patients with weak antiviral responses. These two entities are possibly associated with distinct cognitive and psychiatric disorders. It will therefore be crucial in the future to determine whether patients who experienced severe inflammation in the acute phase and anosognosia of cognitive impairment and fatigue 6–9 months post-infection will follow a course with a high long-term neurodegenerative risk (Mavrikaki et al., 2022; Duff et al., 2025). It will also be necessary to study, at the other end of the spectrum of awareness of disorders, the psychiatric evolution and quality of life of people with severe long-term subjective complaints (Becker et al., 2023).

Importantly, different dimensions of fatigue (physical, cognitive, social, psychological) showed distinct immune associations (Billones et al., 2021; Campos et al., 2022). IFNγ emerged as the most consistent predictor of fatigue, particularly in relation to total and physical fatigue, resembling its role in Epstein-Barr virus-related fatigue syndromes (Bellmann-Weiler et al., 2008). Meanwhile, TNFα was associated with both total and physical fatigue, while IL-1RA appeared more closely linked to fatigue awareness. These findings suggest the need for personalized approaches based on immune profiles.

Subgroup analyses revealed immune–fatigue associations varied by illness severity. Among patients requiring intermediate care, monocyte percentage correlated with total and physical fatigue, whereas inflammatory markers did not significantly predict long-term fatigue (see SI 5–9). Conversely, for intensive care patients, TNFα was linked to total, cognitive, and social fatigue, and both IFNγ and IL-1RA predicted multiple fatigue dimensions. This suggests that while viral load and acute respiratory distress contribute to fatigue, immune dysregulation plays an independent role in post-COVID symptom persistence. Curiously, a pattern seems to emerge regarding neutrophil counts. Neutrophils are positively associated with subjective fatigue in all groups. All other significant markers are negatively associated with subjective fatigue. Neutrophils and associated signaling pathways (e.g., TLR, PAMP, DAMP) could be an interesting target for a better understanding of post-COVID fatigue (Paludan and Mogensen, 2022; Saito et al., 2024).

However, it seems necessary to remain cautious, as measuring subjective fatigue remains a complex task that is difficult to generalize between individuals, and as there is no cut-off point that determines the threshold at which a person is significantly fatigued. Few patients have fatigue with a Z score greater than 1.25 or less than 1. This can be explained by the fact that this scale (EMIF-SEP) is standardized on a population of people with multiple sclerosis. Beyond the statistical aspects related to the cohort, we can note from our observations that some people, after COVID-19 infection (with no previous medical history), may experience clinically higher fatigue than people with multiple sclerosis, regardless of the type of hospitalization in the acute phase. Thus, the fatigue experienced after SARS-CoV-2 infection can lead to significant disability in some patients. Finally, the inflammatory stratification performed did not reveal any intergroup differences. This lack of effect could indicate that differences in post-COVID fatigue symptoms between patients do not result from direct inflammatory mechanisms. Instead, they could be attributable to the direct cytopathogenic effects of SARS-CoV-2 on organs (Maliha et al., 2024),the activation of certain signaling pathways (Paludan and Mogensen, 2022), or the possibility that the inflammatory response was measured too early. In this context, inflammatory mediation analyses could be considered to explore the links between post-COVID cognition or fatigue and vascular phenomena, which are often involved in the persistence of symptoms (Altmann et al., 2023). It would also be relevant to investigate cognitive impairments and associated signaling pathways in detail. Finally, it is essential to study the general population with comorbidities, particularly non-hospitalized patients in the acute phase. Our sample, which consisted only of people with no medical history, is not representative of the general population, which is often affected by various comorbidities.

Finally, we note several limitations of this study. The relatively small sample size, while statistically adjusted, necessitates replication in larger cohorts to improve generalizability. Additionally, as our cohort consisted solely of hospitalized patients, findings may not fully apply to individuals with milder COVID-19 cases. Another challenge is the variability in fatigue measurement; while the EMIF-SEP questionnaire has been used in post-COVID research, additional objective assessments (e.g., VO2 max, actigraphy) would enhance validity. Furthermore, the retrospective nature of our analysis introduces potential confounding factors, including the time elapsed since infection and individual socio-economic vulnerabilities. Although we observed inter-individual variations (expected in the context of COVID-19 (Song et al., 2021)), we do not have replicates available for the quantification of immune markers. This prevents the calculation of intra-individual coefficients of variation and therefore we cannot fully assess the analytical variability of these measures. The observed variability highlights that some patients responded strongly to the infection, while others responded only moderately. This again reflects that several post-COVID patient phenotypes evolve over time between the acute phase and the chronic period. Future research should incorporate longitudinal immune-cognitive assessments and investigate peripheral blood mononuclear cells (PBMCs) for deeper immunological insights. It is also important to note that the interpretation of inflammatory stratifications in relation to post-COVID fatigue status remains an ad hoc hypothesis, formulated in response to the observed findings. As such, it should be considered exploratory and interpreted with caution. Future research using larger, independent samples and explicitly defined hypotheses will be necessary to test and validate this preliminary explanation. Finally, the absence of a SARS-CoV-2-naïve control group complicates interpretation within the broader population. Given the high prevalence of COVID-19 worldwide, recruiting truly uninfected controls remains challenging. Despite these limitations, our results underscore the role of early immune response in long-term fatigue and cognition, and point toward the value of immunological biomarkers for identifying at-risk individuals. Future longitudinal studies integrating immuno-cognitive measures and PBMC profiling are warranted.

## 5 Conclusion

Our study highlights the complex interplay between acute immune markers and post-COVID fatigue. Key inflammatory markers, particularly TNFα, IL-1RA, and IFNγ, appear to influence different dimensions of fatigue and cognitive fatigue awareness months after infection. These findings support a multifaceted model of post-COVID fatigue and cognitive dysfunction, suggesting potential immune-driven subtypes that could inform personalized interventions. Future research should explore the evolution of these fatigue dimensions in relation to socio-economic factors, pre-existing vulnerabilities, and potential neurodegenerative risks, ultimately contributing to optimized post-COVID care strategies.

## CRediT authorship contribution statement

**A. Nuber-Champier:** Writing – review & editing, Writing – original draft, Visualization, Validation, Methodology, Investigation, Formal analysis, Conceptualization. **G. Breville:** Writing – review & editing, Writing – original draft, Methodology, Investigation. **P. Voruz:** Writing – review & editing, Methodology, Investigation. **I. Jacot de Alcântara:** Writing – review & editing, Methodology, Investigation. **P.H. Lalive:** Writing – review & editing, Writing – original draft, Supervision, Resources, Methodology, Investigation. **G. Allali:** Writing – review & editing, Resources, Methodology. **L. Benzakour:** Writing – review & editing. **K.-O. Lövblad:** Writing – review & editing. **O. Braillard:** Writing – review & editing. **M. Nehme:** Writing – review & editing, Methodology. **M. Coen:** Writing – review & editing. **J. Serratrice:** Writing – review & editing. **J.-L. Reny:** Writing – review & editing. **J. Pugin:** Writing – review & editing. **I. Guessous:** Writing – review & editing, Resources, Methodology. **B.N. Landis:** Writing – review & editing. **A. Cionca:** Writing – review & editing, Methodology, Investigation. **F. Assal:** Writing – review & editing, Supervision, Project administration, Funding acquisition, Conceptualization. **J.A. Péron:** Writing – review & editing, Writing – original draft, Validation, Supervision, Resources, Project administration, Methodology, Investigation, Funding acquisition, Conceptualization.

## Data accessibility

The data analyzed and published are available on Yareta at the following address: https://yareta.unige.ch/home/search?search=search %3Dcovid-cog.

## Funding

The present research was supported by 10.13039/100000001Swiss National Science Foundation (10.13039/501100001711SNSF) funding to JAP (10.13039/100006150PI) and FA (Co-10.13039/100006150PI) (grant no. 220041).

## Declaration of competing interest

No conflicts of interest to be declared.

## Data Availability

Data will be made available on request.
