# Supplementary material for: Inflammatory predictors of Post-COVID fatigue
Source: Brain Behav Immun Health. 2025 Sep 17;49:101109. doi: 10.1016/j.bbih.2025.101109 (PMC12506529; doi:10.1016/j.bbih.2025.101109)
Supplement: Multimedia component 1 [file mmc1.docx]

Supplementary information

**SI 1. Socio-demographic and clinical data concerning patients hospitalized in conventional care.**

Table SI 1. Socio-demographic and clinical data from the sample of patients hospitalized in conventional care in the acute phase and assessed 6-9 months after SARS-CoV-2 infection.

|  | Patients hospitalized in conventional care | |
| --- | --- | --- |
|  | *N*= 41 |  |
| Mean age in years (± SD) | 55.68 (± 10.62) | |
| Education level (1/2/3) | 2/10/29 |  |
| Sex (F/M) | 14/27 |  |
| Mean days of hospitalization (± SD) | 12.12 (± 12.68) | |
| Mean days between positive RT-PCR test and collection of immunological data (± SD) | 2.30 (± 3.86) | |
| Diabetes (Yes/No) | 4/37 |  |
| History of respiratory disorders (Yes/No) | 5/36 |  |
| History of cardiovascular disorders (Yes/No) | 7/34 |  |
| History of psychiatric disorders (Yes/No) | 1/40 |  |

*Note.* Education level: 1 = compulsory schooling, 2 = post-compulsory schooling, and 3 = university degree or equivalent. RT-PCR: reverse transcription polymerase chain reaction; SD: standard deviation; Sex F: female and M: mal.

**SI 2. Socio-demographic and clinical data relating to patients hospitalized in intensive care.**

Table SI 2. Socio-demographic and clinical data from the sample of patients hospitalized in intensive care in the acute phase and assessed 6-9 months after SARS-CoV-2 infection.

|  | Patients hospitalized in ICU | |
| --- | --- | --- |
|  | *N*= 24 |  |
| Mean age in years (± SD) | 62.08 (± 12.03) | |
| Education level (1/2/3) | 1/10/13 |  |
| Sex (F/M) | 5/19 |  |
| Mean days of hospitalization (± SD) | 40.13 (± 32.07) | |
| Mean days between positive RT-PCR test and collection of immunological data (± SD) | 1.08 (± 2.62) | |
| Diabetes (Yes/No) | 1/23 |  |
| History of respiratory disorders (Yes/No) | 6/18 |  |
| History of cardiovascular disorders (Yes/No) | 6/18 |  |
| History of psychiatric disorders (Yes/No) | 1/23 |  |

*Note.* Education level: 1 = compulsory schooling, 2 = post-compulsory schooling, and 3 = university degree or equivalent. RT-PCR: reverse transcription polymerase chain reaction; SD: standard deviation; Sex F: female and M: mal.

**SI 3. Immune markers of patients hospitalized in conventional care with COVID-19 on admission to hospital.**

Table SI 3. Immune markers of patients.

| Immune markers | Cytokine count on day 1 of hospitalization (*N*= 17) and monocytes (*N*= 32) Median [95%CI] |
| --- | --- |
| TNFα (pg/ml) | 3.44 [2.57;4.68] |
| IL-1Ra (pg/ml) | 3604.96 [1951.30;4636.80] |
| IFNγ (pg/ml) | 1.65 [.84;1.66] |
| IL-1β (pg/ml) | -0.12 [-.56;-.031] |
| IL-6 (pg/ml) | 4.51 [2.54 ; 19.93] |
| IL-8 (pg/ml) | 10.05 [6.23 ; 22.45] |
| Neutrophils % | 78.05 [65.05 ; 83,08] |
| Neutrophils (G/l) | 5.25 [4.10 ; 7.72] |
| Eosinophils % | 0 [-.004 ; .237] |
| Eosinophils (G/l) | 0 [0 ; .01] |
| Basophils % | .10 [.035 ; .24] |
| Basophils (G/l) | .008 [.003 ; .014] |
| Monocytes (G/l) | .46 [.28 ; .62] |
| Monocytes % | 7.45 [3.91;9.54] |

*Note.* IFNγ: interferon gamma; IL: interleukin; TNFα: tumor necrosis factor.

**SI 4. Immune markers of patients hospitalized in intensive care with COVID-19 on admission to hospital.**

Table SI 4. Immune markers of ICU patients.

| Immune markers | Cytokine count on day 1 of hospitalization (*N*= 22) and monocytes (*N*= 22) Median [95%CI] |
| --- | --- |
| TNFα (pg/ml) | 4.07 [3.52;6.65] |
| IL-1Ra (pg/ml) | 6256.74 [5983.11100.29] |
| IFNγ (pg/ml) | 1.41 [0.81;2.93] |
| IL-1β (pg/ml) | 0.49 [0.33;1.22] |
| IL-6 (pg/ml) | 23.40 [19.43 ; 47.75] |
| IL-8 (pg/ml) | 14.93 [11.32 ; 26.95] |
| Neutrophils % | 75.05 [71.18 ; 82.02] |
| Neutrophils (G/l) | 4.92 [4.18 ; 5.93] |
| Eosinophils % | 0 [-.013 ; .62] |
| Eosinophils (G/l) | 0 [-.001 ; .046] |
| Basophils % | .20 [.13 ; .35] |
| Basophils (G/l) | .01 [.008 ; .021] |
| Monocytes (G/l) | .34 [.28 ; .46] |
| Monocytes % | 6.35 [4.65;7.84] |

*Note.* IFNγ: interferon gamma; IL: interleukin; TNFα: tumor necrosis factor.

**SI 5. Association and predictability of subjective fatigue measured 6-9 months post-infection as a function of acute immunity in patients hospitalized in conventional care.**

*Spearman correlation*

We observed a significant association between cognitive fatigue scores and the percentage of monocytes (*p*=.029 ; *r*=-0.38) and a significant association between psychological fatigue scores and the percentage of monocytes (*p*=.018 ; *r*=-0.41). Neutrophils count were associated with cognitive fatigue scores and psychological fatigue scores (*r=.*70*; p=.*010*, r*=.65, *p*= .022 respectively).

*Generalized linear mixed model*

We performed GLMM models based on significant correlations. Neutrophil counts were found to be a significant predictor of cognitive and psychological fatigue (*F* =6.46 ; *t*=2.54; *p*=.029 and *F* =7.55; *t*=2.74; *p*=.021).

**SI 6. Association and predictability of subjective fatigue measured 6-9 months post-infection as a function of acute immunity in intensive care hospital patients.**

*Spearman correlation*

Concerning total fatigue, we observe a significant association with levels of acute TNFα (*p*=.008 ; *r*=-0.54). Acute TNFα and IL-1RA levels were also associated with cognitive fatigue scores (*p*=.001 ; *r*=-0.65) et (*p*=.013 ; *r*=-0.52). Acute TNFα levels were associated with social fatigue scores (*p*=.039 ; *r*=-0.44).

Acute IL-6 levels were associated with total fatigue scores, physical fatigue scores and social fatigue scores (*p=.*034, *r=*-.45*; p=.*017*, r=-.*50*; p=.*035, *r*=-.41, respectively). Finally, neutrophil percentage and neutrophils count were associated with total fatigue scores (*r=.*44*; p=.*048*, r*=.48, *p*= .032 respectively) and physical fatigue scores ((*r=.*57*; p=.*008*, r*=.48, *p*= .028 respectively).

*Generalized linear mixed model*

- The total fatigue prediction model revealed that IL-1RA levels (*F* = 6.92; *p* =.020; 95%CI [-1.25; -0.12]) and IFNγ (*F* = 10.18; *p* =.007; 95%CI [0.15; 0.78]) could predict total fatigue. The percentage of neutrophils was a significant predictor of total fatigue scores (*F*=4.49; *t*=2.11; *p*=.048).
- Cognitive fatigue prediction model revealed that IL-1RA levels (*F* = 8.16; *p* =.013; 95%CI [-1.33; -0.19]) and IFNγ (*F* = 7.42; *p* =.016; 95%CI [0.085; 0.71]) could predict cognitive fatigue.
- The physical fatigue prediction model revealed that IFNγ levels (*F* = 7.73; *p* =.015; 95%CI [0.13; 1.03]) could predict these scores. Neutrophil counts were a predictor of physical fatigue scores (*F*=4.60; *t*=2.14; *p*=.046).
- The social fatigue prediction model revealed that IL-1RA levels (*F* = 11.71; *p* =.004; 95%CI [-1.14; -0.32]) and IFNγ levels (*F* = 10.92; *p* =.005; 95%CI [0.17; 0.79]) could predict social fatigue.
- There were no significant results for psychological fatigue.

**SI 7. Association and prediction of cognitive fatigue awareness according to acute hospitalization subgroups.**

Awareness of fatigue was neither associated with nor predicted by immunity when considering acute hospitalisation subgroups.

There were no significant results for Spearman correlations or linear regressions.

**SI 8. Association between plasma cytokines concentration, monocytes % measured in the acute phase of COVID-19 and psychological and social fatigue 6-9 months post-infection.**

*Psychological*

The relationships between the percentage of monocytes in the blood, the concentration of TNFα and psychological fatigue were not statistically significant but had values closest to the threshold at .05, i.e. (*p*=.052 ; *r*=-0.26) and (*p*=.06 ; *r*=-0.31).

*Social*

The relationships between IL-1RA, TNFα concentration and social fatigue were not statistically significant after FDR correction but had values closest to the threshold at .05, namely (*p*=.063 ; *r*=-0.30) and (*p*=.043 ; *r*=-0.33).

**SI 9. Prediction of cognitive, social and psychological fatigue 6-9 months post-infection by inflammatory response measured during the acute phase of SARS-CoV-2 infection.**

*Cognitive fatigue*

The concentration of plasma IL-1RA in the cognitive fatigue prediction model is the parameter closest to statistical significance, i.e. (*F* = 3.18; *p* =.086; 95%CI [-0.83; 0.60]).

*Social fatigue*

The concentration of plasma IL-1RA and IFNγ in the social fatigue prediction model were the parameters closest to statistical significance, i.e. (*F* = 4.06; *p* =.054; 95%CI [-0.77; 0.008]) and (*F* = 3.86; *p* =.060; 95%CI [-0.036; 0.61]).

*Psychological fatigue*

The concentration of plasma IL-1RA in the psychological fatigue prediction model is the parameter closest to statistical significance, i.e. (*F* = 2.28; *p* =.14; 95%CI [-0.92; 0.14]).

**SI 10. Details of groups by fatigue sub-dimension**

*Total fatigue*

Thirty-seven patients completed the EMIF-SEP questionnaire. Among them, 26 presented fatigue within the normal range (z-score between -1 and 1.25 inclusive), nine presented fatigue below the threshold z-score of -1, and two presented z-scores greater than 1.25.

*Cognitive fatigue*

Thirty-five patients had normal fatigue (z-score between -1 and 1.25 inclusive), two had z-scores above 1.25.

*Physical fatigue*

Twenty-five patients had normal fatigue (z-score between -1 and 1.25 inclusive), 12 had z-scores below -1.

*Social fatigue*

Thirty-two patients had normal fatigue (z-score between -1 and 1.25 inclusive), three had z-scores below -1 and two above 1.25.

*Psychological fatigue*

Sixteen patients had normal fatigue (z-score between -1 and 1.25 inclusive), 20 had z-scores below -1 and one had a z-score above 1.25.

**SI 11. Inflammatory differences between the different fatigue groups obtained by z-score.**

The results of intergroup differences in inflammatory concentrations obtained by subgroups according to different dimensions of fatigue are presented below.

Although some results are significant, their values must be weighted heavily given the limited number of patients in certain groups. However, based on norms for a population with multiple sclerosis, these results provide insight that can be used to grade the fatigue experienced by post-COVID patients.

No significant differences were found between fatigue groups based on type of hospitalisation in the acute phase (total fatigue: *p*=.18; cognitive fatigue: *p*=.07; physical fatigue: *p*=.18; psychological fatigue: *p*=.46; social fatigue: *p*=.21).

*Total fatigue*

|  | TNF | IL6 | IL-8 | IL-1ra | Il-1B | IFN | neutrophils % | neutrophils (G/l) | eosinophils % | eosinophils (G/l) | basophils % | basophils (G/l) | monocytes % | Monocytes (G/l) |
| --- | --- | --- | --- | --- | --- | --- | --- | --- | --- | --- | --- | --- | --- | --- |
| H Kruskal-Wallis | 3,12 | ,46 | 4,97 | 2,88 | ,79 | 4,51 | 2,55 | 5,93 | ,86 | ,62 | ,38 | 2,54 | 2,88 | 6,22 |
| df | 2 | 2 | 2 | 2 | 2 | 2 | 2 | 2 | 2 | 2 | 2 | 2 | 2 | 2 |
| p-value | ,21 | ,79 | ,08 | ,23 | ,67 | ,10 | ,27 | ,05 | ,64 | ,73 | ,82 | ,28 | ,23 | ,04 |

**Note.** IFNγ: interferon gamma; IL: interleukin; TNFα: tumor necrosis factor.

*Cognitive fatigue*

|  | TNF | IL6 | IL-8 | IL-1ra | Il-1B | IFN | neutrophils % | neutrophils (G/l) | eosinophils % | eosinophils (G/l) | basophils % | basophils (G/l) | monocytes % | Monocytes (G/l) |
| --- | --- | --- | --- | --- | --- | --- | --- | --- | --- | --- | --- | --- | --- | --- |
| H Kruskal-Wallis | 2,95 | ,22 | 4,91 | 2,82 | ,46 | ,34 | ,09 | 4,09 | ,05 | ,21 | ,22 | 2,46 | ,54 | 5,47 |
| df | 1 | 1 | 1 | 1 | 1 | 1 | 1 | 1 | 1 | 1 | 1 | 1 | 1 | 1 |
| *p*-value | ,08 | ,63 | ,02 | ,09 | ,49 | ,55 | ,75 | ,04 | ,81 | ,64 | ,63 | ,11 | ,45 | **,01** |

**Note.** IFNγ: interferon gamma; IL: interleukin; TNFα: tumor necrosis factor.

We observe significant differences in IL-8, neutrophil count and monocyte count. However, after FDR correction, only the monocyte count remains significant (*p*=.01). This result should be weighted in view of the number of people in the different groups (*n*=35 in the normal range and *n*=2 above a z-score of 1.25).

*Physical fatigue*

|  | TNF | IL6 | IL-8 | IL-1ra | Il-1B | IFN | neutrophils % | neutrophils (G/l) | eosinophils % | eosinophils (G/l) | basophils % | basophils (G/l) | monocytes % | Monocytes (G/l) |
| --- | --- | --- | --- | --- | --- | --- | --- | --- | --- | --- | --- | --- | --- | --- |
| H Kruskal-Wallis | 3,32 | 2,42 | ,09 | ,28 | ,07 | 1,24 | 2,51 | 4,46 | ,41 | ,17 | 4,06 | 1,88 | 3,27 | ,04 |
| df | 1 | 1 | 1 | 1 | 1 | 1 | 1 | 1 | 1 | 1 | 1 | 1 | 1 | 1 |
| *p*-value | ,06 | ,11 | ,75 | ,59 | ,78 | ,26 | ,11 | ,03 | ,52 | ,67 | ,04 | ,17 | ,070 | ,83 |

**Note.** IFNγ: interferon gamma; IL: interleukin; TNFα: tumor necrosis factor.

We observe no significant differences after FDR correction.

*Social fatigue*

|  | TNF | IL6 | IL-8 | IL-1ra | Il-1B | IFN | neutrophils % | neutrophils (G/l) | eosinophils % | eosinophils (G/l) | basophils % | basophils (G/l) | monocytes % | Monocytes (G/l) |
| --- | --- | --- | --- | --- | --- | --- | --- | --- | --- | --- | --- | --- | --- | --- |
| H Kruskal-Wallis | 3,47 | 2,64 | 6,37 | 4,30 | ,80 | 4,52 | ,57 | 4,59 | ,26 | ,46 | ,27 | 2,46 | 2,57 | 6,75 |
| df | 2 | 2 | 2 | 2 | 2 | 2 | 2 | 2 | 2 | 2 | 2 | 2 | 2 | 2 |
| p-value | ,17 | ,26 | ,04 | ,11 | ,67 | ,10 | ,75 | ,10 | ,87 | ,79 | ,87 | ,29 | ,27 | **,03** |

**Note.** IFNγ: interferon gamma; IL: interleukin; TNFα: tumor necrosis factor.

We observe no significant differences after FDR correction.

*Psychological fatigue*

|  | TNF | IL6 | IL-8 | IL-1ra | Il-1B | IFN | neutrophils % | neutrophils (G/l) | eosinophils % | eosinophils (G/l) | basophils % | basophils (G/l) | monocytes % | Monocytes (G/l) |
| --- | --- | --- | --- | --- | --- | --- | --- | --- | --- | --- | --- | --- | --- | --- |
| H Kruskal-Wallis | 1,44 | ,30 | 2,84 | 1,21 | ,72 | 3,61 | 1,30 | 4,99 | ,45 | ,42 | ,29 | 1,25 | 3,27 | 3,38 |
| df | 2 | 2 | 2 | 2 | 2 | 2 | 2 | 2 | 2 | 2 | 2 | 2 | 2 | 2 |
| p-value | ,48 | ,85 | ,24 | ,54 | ,69 | ,16 | ,52 | ,08 | ,76 | ,81 | ,86 | ,53 | ,19 | ,18 |

**Note.** IFNγ: interferon gamma; IL: interleukin; TNFα: tumor necrosis factor.

We observe no significant differences after FDR correction.

**SI 12. Intergroup results of subjective fatigue differences related to inflammation stratification**

We determined that 20 people had low inflammation according to our calculation (Z<1); seven people had moderate inflammation (-1<Z>1) and 10 people had high inflammation (Z>1).

We did not observe any significant differences between these groups in terms of all dimensions of fatigue (total fatigue: H=3.45, *p*=.17; cognitive fatigue: H=1.79, *p*=.40; physical fatigue: H= 2.22, *p*=.32; psychological fatigue: H= 1.98, *p*=.37; social fatigue: H=2.60, *p*=.27).

**SI 13. Associations between IL-6/IL-8, non-monocyte leukocytes markers and subjective fatigue scores.**

We observed that neutrophil counts were positively associated with cognitive fatigue after FDR correction regarding leukocyte distribution, although ± 2 patients for these markers showed a lack of behavioural fatigue data linked to the presence of immune markers. However, we observed a significant negative correlation between IL-6 levels and physical fatigue after FDR correction. Only neutrophil count was found to be significant in GLMM predictive models for cognitive fatigue.

*Spearman correlations :*

|  |  | IL-6 | IL-8 |
| --- | --- | --- | --- |
| *Total fatigue* | Correlation coefficient | -,244 | -,173 |
|  | *p*- value | ,145 | ,305 |
|  | *N* | 37 | 37 |
| *Cognitive fatigue* | Correlation coefficient | -,035 | -,249 |
|  | *p*- value | ,839 | ,137 |
|  | *N* | 37 | 37 |
| *Physical fatigue* | Correlation coefficient | **-,335*** | -,118 |
|  | *p*- value | **,043** | ,486 |
|  | *N* | 37 | 37 |
| *Social fatigue* | Correlation coefficient | -,242 | -,182 |
|  | *p*- value | ,150 | ,280 |
|  | *N* | 37 | 37 |
| *Psychological fatigue* | Correlation coefficient | -,031 | -,192 |
|  | *p*- value | ,856 | ,254 |
|  | *N* | 37 | 37 |
| Cognitive fatigue awareness | Correlation coefficient | -,061 | ,108 |
|  | *p*- value | ,735 | ,551 |
|  | *N* | 33 | 33 |

|  |  | | Neutrophils (G/l) | Neutrophils % | Eosinophils % | Eosinophils (G/l) | Basophils % | Basophils (G/l) | Monocytes (G/l) |
| --- | --- | --- | --- | --- | --- | --- | --- | --- | --- |
| *Total fatigue* | | Correlation coefficient | ,267 | ,173 | -,037 | -,005 | -,032 | ,061 | -,095 |
|  |  | *p*- value | ,049 | ,206 | ,788 | ,970 | ,817 | ,657 | ,486 |
|  |  | *N* | 55 | 55 | 54 | 56 | 54 | 55 | 56 |
| *Cognitive fatigue* | | Correlation coefficient | **,332** | ,170 | -,054 | -,008 | -,049 | ,034 | -,098 |
|  |  | *p*- value | **,013** | ,215 | ,700 | ,953 | ,726 | ,803 | ,472 |
|  |  | *N* | 55 | 55 | 54 | 56 | 54 | 55 | 56 |
| *Physical fatigue* | | Correlation coefficient | ,237 | ,175 | ,041 | ,058 | -,059 | ,042 | -,082 |
|  |  | *p*- value | ,082 | ,200 | ,768 | ,672 | ,673 | ,763 | ,548 |
|  |  | *N* | 55 | 55 | 54 | 56 | 54 | 55 | 56 |
| *Social fatigue* | | Correlation coefficient | ,166 | ,109 | -,044 | -,006 | ,066 | ,139 | -,017 |
|  |  | *p*- value | ,225 | ,428 | ,750 | ,966 | ,635 | ,313 | ,900 |
|  |  | *N* | 55 | 55 | 54 | 56 | 54 | 55 | 56 |
| *Psychological fatigue* | | Correlation coefficient | ,294 | ,082 | -,076 | -,048 | -,007 | ,063 | -,070 |
|  |  | *p*- value | ,030 | ,553 | ,586 | ,727 | ,958 | ,646 | ,610 |
|  |  | *N* | 55 | 55 | 54 | 56 | 54 | 55 | 56 |
| Cognitive fatigue awareness | | Correlation coefficient | ,245 | ,062 | -,110 | -,076 | -,023 | ,029 | -,030 |
|  |  | *p*- value | ,093 | ,674 | ,462 | ,607 | ,879 | ,846 | ,840 |
|  |  | *N* | 48 | 48 | 47 | 48 | 47 | 48 | 49 |

*Generalized linear mixed model :*

*Total fatigue*

The models predicting total fatigue using IL-6 showed the following results (β = -0.11; t = 1.24; p = .27); on the other hand, the models using IL-8 as a predictor revealed the following results (β = -.24; F =4.13 ; *p* =.50).

*Cognitive fatigue*

The models predicting total fatigue using IL-6 showed the following results (β =.001 ; *F* =.00 ; *p* =.99); on the other hand, the models using IL-8 as a predictor revealed the following results (β = -13.38; *F* =3.42 ; *p* =.07).

*Physical fatigue*

The models predicting total fatigue using IL-6 showed the following results (β =-.21 ; F =3.50 ; *p* = .069); on the other hand, the models using IL-8 as a predictor revealed the following results (β = -.18; *F* =2.13 ; *p* =.15).

*Social fatigue*

The models predicting total fatigue using IL-6 showed the following results (β = -.11; *F* = 1.36; *p* =.25 ); on the other hand, the models using IL-8 as a predictor revealed the following results (β = -.20; *F* = 2.73 ; *p* =.10).

*Psychological fatigue*

The models predicting total fatigue using IL-6 showed the following results (β = -.003; *F* =.001 ; *p* =.98); on the other hand, the models using IL-8 as a predictor revealed the following results (β = -.29; *F* =3.32 ; *p* =.07).

In order to limit the number of analyses, we created GLMM models based on significant markers in terms of correlations, i.e., neutrophil counts in relation to cognitive fatigue.

*Cognitive fatigue*

Models predicting cognitive fatigue based on neutrophil counts revealed the following significant results (β = .05; *F* = 8.97; *p* = **.004**).
